# Zuranolone therapy protects frontal cortex neurodevelopment and improves behavioral outcomes after preterm birth

**DOI:** 10.1002/brb3.70009

**Published:** 2024-09-05

**Authors:** Roisin A. Moloney, Hannah K. Palliser, Carlton L. Pavy, Julia C. Shaw, Jonathan J. Hirst

**Affiliations:** ^1^ School of Biomedical Sciences and Pharmacy University of Newcastle Newcastle Australia; ^2^ Hunter Medical Research Institute Mothers and Babies Research Centre Newcastle Australia

**Keywords:** behavior, dopamine, GABA, neurosteroid, preterm birth

## Abstract

**Background:**

Preterm birth is associated with brain injury and long‐term behavioral abnormalities, for which there are limited prevention options. When born preterm, infants prematurely lose placental neurosteroid (allopregnanolone) support. This increases the risk of excitotoxic damage to the brain, which increases the risk of injury, causing long‐term deficits in behavior, myelination, and alterations to neurotransmitter pathways. We propose that postnatal restoration of neurosteroid action through zuranolone therapy will reduce neurological impairments following preterm birth.

**Methods:**

Guinea pig dams underwent survival cesarean section surgery to deliver pups prematurely (GA64) or at term (GA69). Between birth and term equivalence age, preterm pups received vehicle (15% β‐cyclodextrin) or the allopregnanolone analogue zuranolone (1 mg/kg/day). Behavioral analysis was performed at postnatal day (PND) 7 and 40, before tissue collection at PND 42. Immunostaining for myelin basic protein (MBP), as well as real‐time polymerase chain reaction to characterize oligodendrocyte lineage and neurotransmitter pathways, was performed in frontal cortex tissues.

**Results:**

Zuranolone treatment prevented the hyperactive phenotype in preterm‐born offspring, most markedly in males. Additionally, preterm‐related reductions in MBP were ameliorated. Several preterm‐related alterations in mRNA expression of dopaminergic, glutamatergic, and GABAergic pathways were also restored back to that of a term control level.

**Conclusion:**

This is the first study to assess zuranolone treatment as a neuroprotective therapy following preterm birth. Zuranolone treatment improved behavioral outcomes and structural changes in the preterm offspring, which continued long term until at least a late childhood timepoint. Clinical studies are warranted for further exploring the neuroprotective possibilities of this treatment following preterm birth.

## Graphical abstract

Preterm‐born guinea pigs displayed hyperactive behavior, reduced myelin, and disrupted neurotransmitter pathway expression at late childhood equivalence. Treatment with zuranolone in the immediate postnatal period following preterm birth was able to prevent these deficits from occurring and restore behavioral abnormalities to a term‐born phenotype.

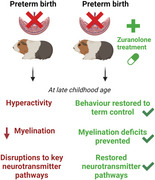

## 1 INTRODUCTION

Preterm birth is one of the major causes of neurodevelopmental disorders globally (Soleimani et al., 2014). In recent years with advancements in neonatal care, the mortality rate of preterm‐born infants has reduced (Wilson‐Costello et al., 2005). However, infants that survive have an increased risk of a range of neurological disorders, including cerebral palsy (Arnaud et al., 2021), schizophrenia (Vanes et al., 2022), autism (Crump et al., 2021), and in particular for moderate‐late preterm birth; attention deficit hyperactivity disorder (ADHD) (Fraiman et al., 2023; Harris et al., 2013; Rommel et al., 2017). ADHD is a relatively common disorder in children that often does not become apparent until school age. There are currently limited targeted treatments for the prevention of this condition, and the resulting economic and social burden on the individual are substantial (Schein et al., 2022).

This increase in neurodevelopmental disorders following preterm birth is due to the critical stage of neurodevelopment that is occurring at this stage, as the brain is particularly vulnerable to damage in the last trimester of gestation (Bouyssi‐Kobar et al., 2016). This is due not only to the premature exposure to the stimulating ex utero environment, but also to the premature loss of the placentally derived neurosteroid allopregnanolone, and the subsequent significantly reduced exposure of the trophic effects of this neurosteroid compared to term‐born counterparts. Allopregnanolone (3α‐hydroxy,5α‐pregnane‐20‐one) is found in remarkably increased concentrations in the fetal brain and plasma compared to post birth (Bicíková et al., 2002; Kelleher et al., 2013). This neurosteroid allosterically modulates GABA_A_ receptors, increasing inhibitory tone, which is critical for later stages of brain development such as myelination. In the human and guinea pig, GABAergic stimulation produces an excitatory action in early gestation. However, it undergoes a switch to produce an inhibitory response, which occurs at approximately 0.6 of gestation, which is necessary for key events in development such as the sleep‐like states (Coleman et al., 2013; Sedmak et al., 2016). Following preterm birth, the premature loss of placental support and a reduction of this neuroprotective inhibition exposes the developing infant to over‐stimulating, excitotoxic insults (Pregnolato et al., 2019). We have previously highlighted the importance of allopregnanolone through our work using the clinically relevant guinea pig model, showing that preterm‐born offspring have long‐term reductions in myelin basic protein (MBP) in the hippocampus and cerebellum (Shaw et al., 2018, 2015, 2022). Clinical studies have shown preterm birth is associated with marked white matter injury (Agut et al., 2020). It is well established that deficits in white matter microstructure are associated with neurodevelopmental disorders, particularly ADHD (Versace et al., 2021). Therefore, the premature loss of the neuroprotective allopregnanolone and subsequent white matter alterations associated with preterm birth remain an important target for preventative treatments. Preterm birth has also been found to cause alterations to key neurotransmitter pathways that are implicated in neurobehavioral disorders such as ADHD. In our preliminary studies, we have previously shown that guinea pig offspring born moderate‐late preterm have long‐lasting alterations to both the dopaminergic and noradrenergic pathways, both of which are critical in cognitive and attentional control (Moloney et al., 2024). These alterations may also be occurring as a result of the premature loss of neurosteroid and subsequent excitotoxicity, as it is widely known that dopaminergic neurons are particularly sensitive to glutamatergic toxicity (Wang et al., 2020).

Despite these known deficits, little progress has been made in therapeutic options for preterm‐born offspring to prevent these neurological deficits. Magnesium sulfate is a neuroprotective therapy that has shown promising results for protecting preterm‐born infants. However, this requires antenatal treatment, which can be restricting as >50% of preterm births are spontaneous (Menon, 2008). Term‐born infants with hypoxic‐ischemic encephalopathy have shown improvements to neurodevelopmental impairment with clinical therapeutic hypothermia (Natarajan et al., 2018). Preterm‐born infants, however, are developmentally immature with vascular fragility and impaired cerebral autoregulation, limiting the use of this treatment (Herrera et al., 2018).

Recent studies support the use of allopregnanolone replacement approaches. Allopregnanolone has been shown to reduce apoptosis and improve learning and memory in a rat model of traumatic brain injury (Djebaili et al., 2005). The development of allopregnanolone analogues with increase in half‐life now increases the potential for replacement approaches (Timby et al., 2006). Additionally, allopregnanolone has been shown to readily metabolize back into its precursor, 5α‐dihydroprogesterone, which has a far lower affinity for the GABA_A_ receptor (Liang & Rasmusson, 2018). Zuranolone is a recently developed allopregnanolone analogue that is currently undergoing clinical trials for adult disorders such as anxiety (Marecki et al., 2023) and parkinsonian tremor (Bullock et al., 2021). Zuranolone has recently been FDA approved for as a new post‐birth treatment for post‐partum depression (PPD) (Barnes et al., 2023). This treatment also has the added benefit of being able to be administered orally due to its increased bioavailability (Cha et al., 2024). In our preliminary studies, we have previously shown using in vitro prenatal insult model of oxygen‐glucose deprivation in primary neurons and oligodendrocytes that treatment with zuranolone was able to prevent cytotoxicity and protect neurons and oligodendrocytes from damage (Moloney et al., 2024). However, there is currently no information on the efficacy of zuranolone for neuroprotection in the newborn following preterm birth. We propose that zuranolone treatment in the immediate postnatal period will prevent adverse alterations in key neurotransmitter systems, behavior, and myelination in a clinically relevant long‐gestation species. The aim of the present study is to determine the beneficial effects of postnatal zuranolone therapy on neurodevelopment and the incidence of related neurobehavioral disorders in a guinea pig model of preterm birth. A further aim was to determine effects on key neurotransmitter pathways, which may be involved in the increased association of preterm delivery and mental health disorders.

## 2 METHODS

Unless otherwise stated, all chemicals and reagents were obtained from Sigma Aldrich (Castle Hill, NSW, Australia).

### 2.1 Animals

All animal experiments and procedures in this study were conducted in accordance with the National Health and Scientific Research Council Australian Code of Practice for the Care and Use of Animals for Scientific Purposes and approved by the University of Newcastle Animal Care and Ethics Committee under ethics protocol A‐2021‐102. Tri‐colored outbred female guinea pig dams were obtained from the University of Newcastle Animal Services Unit. Dams were housed indoors under a 12‐h light‐dark cycle and were supplied with a diet of commercial guinea pig pellets, hay, and fresh vegetables. Time‐mated pregnant dams were allocated to either preterm (*n* = 11 dams) or term (*n* = 8 dams).

#### 2.1.1 Cesarean section delivery and post‐operative care of dam

Pregnant dams allocated to preterm cesarean section (c‐section) delivery were administered betamethasone (1 mg/kg subcutaneously; Celestone Chronodose, Merck Sharp & Dohme) on GA62 and GA63 to assist with maturation of the preterm fetal lungs. On the day of surgery, preterm dams (GA64, designated corrected PND‐6) and term dams (GA69, designated corrected PND‐1, with term being GA70/PND0) were fasted for 2 h. Dams were administered atropine (0.05 mg/kg subcutaneously; Atrosite, Troy Lab) to reduce salivary secretions 30 min prior to surgery. Dams were a with isoflurane (1%–3%) via chamber inhalation until unconscious, before moving to the surgery table on a heat pad, with anesthesia maintained through mask inhalation (reduced dose). The surgery site (abdomen) was then prepared by shaving and washing thoroughly with a betadine surgical scrub (iodine 7.5% w/w). Withdrawal and palpebral reflexes of the dam were assessed, and surgical site wiped with 70% ethanol. The remainder of the surgery was performed under aseptic conditions. Lidocaine (2 mg/kg; Lignocaine, Troy Lab) and bupivacaine (1 mg/kg; Marcaine, AstraZeneca) were administered to the incision site as a local anesthetic. A 6 cm incision was made in skin lateral to the midline, ensuring the uterus and organs below were not damaged. A 4 cm incision was made in the uterine wall, and each fetus within its amniotic sac was exteriorized through the myometrial incision, with each placenta gently removed from the myometrial wall. Following suturing of incisions, each dam recovered on a heat pad with blankets. Buprenorphine (0.05 mg/kg subcutaneously; Temvet, Ilium, Troy Lab) was administered immediately pre‐operatively and repeated at 10 and 24 h post operation. At 1 and 24 h post‐operatively, meloxicam (0.1 mg/kg orally; Apex Meloxicam, Dechra) was administered. Dams were returned to home cages when they regained normal posture (within 15 min) and were moving and eating 6–8 h post‐surgery. Lactation commenced 1–4 days post‐surgery and sutures were removed 10 days post‐surgery.

#### 2.1.2 Neonate care

Resuscitation and respiratory support of preterm and term pups occurred as previously described (Berry et al., 2015; Shaw et al., 2016). Briefly, following removal from the amniotic sac, each pup was dried and stimulated by rubbing the chest wall to encourage spontaneous respiratory effort. Respiratory support was provided by continuous positive airway pressure (CPAP) at 5 cm H_2_O using the “Neopuff” T‐piece infant resuscitator (Fisher & Paykel) with blended medical air and oxygen at 5L/min for approximately 5 min. If spontaneous respiration was not achieved, positive pressure ventilation at 60 breaths/min was provided. Curosurf (poractant alfa, 240 mg surfactant/3 mL) was administered nasally (80 uL) immediately post birth and at 3 h. Once stable, preterm pups were housed in a warm humidified incubator (Bird Brooder ICU, Bell South) for approximately 48–72 h, and from there they were returned to their mother's cage. Term pups were returned to home cages after a maximum of 24 h.

Preterm pups received 0.2–0.5 mL of Impact guinea pig colostrum replacement (Wombaroo Food Products) every 2 h until 12 h old. From there, pups received Impact guinea pig milk replacement (Wombaroo Food Products) every 2 h or until independent feeding occurred. Preterm dams commenced lactation at approximately PND4, with supplemental feeding following this varying based on weight and need. Pups assigned to the preterm zuranolone‐treated group received 1 mg/kg zuranolone orally in 1 μL/µg 15% β‐cyclodextrin vehicle twice daily until term equivalence. Pups assigned to the preterm vehicle group received 1 μL/µg 15% β‐cyclodextrin orally until term equivalence. Term pups also received colostrum and milk replacement feeding; however, were able to feed off their mothers by PND2 and required less supplemental feeding. Preterm pups (male and female, vehicle and zuranolone treated) were fed approximately 3–6 mL on days PND0‐2, 10 mL daily for PND3‐5, and 0–5 mL daily for PND4‐8. Term pups (male and female) were fed 5–7 mL daily PND0‐2 and 0–3 mL daily PND3‐5. All pups received 0.5 mL saline injections subcutaneously at 6, 12, and 18 h post birth to assist with hydration. Wellbeing scores were assessed and recorded every 2 h (refer to Table 1; Shaw et al., 2019). Pups were housed with their littermates and dams until weaning at corrected PND21. Survival rates for term pups were 90% (males) and 100% (females) preterm receiving vehicle were 57% (males) and 77% (females), while preterm receiving zuranolone were 43% (males) and 77% (females).

**TABLE 1:** Wellbeing scores scoring for each category was obtained every 2 h for each pup. Each category was assigned a score from 1 to 4 (with 1 being the poorest and 4 being optimal). Scores were added to give a total out of 12.

Score	Respiration	Posture	Movement
1	Gasping only	Lying down; spasticity of neck and front legs	Only when stimulated
2	Combination of irregular and normal breathing with some gasping	Can sit up, but difficulty in holding head up	Some spontaneous movement
3	Some irregular or shallow breathing (no gasping)	Upright, sitting, can walk but wobbly and uncoordinated	Some activity
4	Normal breathing	Stand and walk easily	Very active and alert

### 2.2 Tissue collection

All pups were euthanized via CO_2_ asphyxiation at corrected PND42 (Shaw et al., 2016). Brains were dissected down the sagittal plane to split the hemispheres, with each left hemisphere fixed in a 10% formalin neutral‐buffered solution for immunohistochemistry, and the frontal cortex from the right hemisphere snap frozen in liquid nitrogen for real‐time polymerase chain reaction (RT‐PCR) analysis.

### 2.3 Behavioral tests

#### 2.3.1 Open field arena

At PND7 and PND40, pups underwent behavioral assessment as previously described (Crombie et al., 2021; Shaw et al., 2019). Open field and elevated plus maze tests were recorded and analyzed using ANY‐maze tracking soft v4.7 (Stoelting Co.). guinea pigs were allowed to explore the open field arena (50 cm × 50 cm) for 5 min and activity was recorded. The recorded image was divided into 5 cm × 5 cm grids and an inner‐zone designated. Parameters quantified included overall distance traveled, number of gridlines crossed entries into the inner zone, distance, and time in the inner zone. The arena was cleaned with 70% ethanol between each animal.

#### 2.3.2 Elevated plus maze

The elevated plus maze apparatus consisted of a cross shaped arena with two open arms and two closed arms (10 cm wide, 50 cm high), as well as a small central arena, elevated 80 cm above the ground (San Diego Instruments). Parameters assessed included time, distance, and entries into either the open or closed arms over a 5‐min testing period.

#### 2.3.3 Startle response system

Acoustic startle response (ASR) and prepulse inhibition of startle response (PPI) were tested in the SR‐Lab Startle Response System (San Diego Instruments). Pups were placed in clear Plexiglass cylinders that allowed them to move but not turn around. These cylinders were mounted on piezoelectric transducers, which detected and recorded movement (startle response). The cylinders were placed in the sound‐attenuated, illuminated, and ventilated response chamber. The startle session began with a 5‐min acclimation period of 70 dB white noise, which continued throughout the session followed by seven blocks of trials. Blocks consisted of pulse alone (120 dB, 30 ms) and varying prepulse (3–20 dB above background, 30 ms with 70 ms interstimulus interval) + pulse trials. Intertrial interval ranged between 8 and 22 s. ASR, PPI, latency, and habituation were assessed.

### 2.4 Immunohistochemistry

To characterize myelination, immunostaining with the mature myelination marker (MBP) was performed as previously described (Shaw et al., 2019). Paraffin embedded brains were cut into 8 µm sections using a Leica RM2145 microtome (Leica Microsystems Pty. Ltd.). Briefly, sections were dewaxed and rehydrated by incubation in xylene and ethanol before antigen retrieval occurred in citrate buffer for 25 min (pH 6.0; 90°C–95°C). Blocking of endogenous peroxidases was achieved by incubation in phosphate‐buffered saline (PBS) containing 3% hydrogen peroxide for 20 min, followed by a goat serum block (2% goat serum, 0.4% BSA, 0.3% Triton‐X in PBS for 1 h) to block non‐specific staining. Slides were incubated in the primary antibody overnight at room temperature (1:1000, M9394), before a 1 h room temperature incubation in the secondary antibody (goat anti‐rat 1:300; B7139). Tertiary antibody incubation occurred for 1 h in streptavidin‐biotin‐horseradish peroxidase complex (ab7403, Abcam) at 1:400 dilution. Immunostaining then occurred via incubation in 3,3′‐diaminobenzidine tetrahydrochloride solution (Metal Enhanced DAB Substrate Kit; Santa Cruz Biotechnology). Sections were imaged using the Aperio imaging system at 20× magnification (Leica Biosystems). Myelination was quantified by relative area coverage (%) of positive staining in cingulate and motor cortices of the caudal frontal cortex with three serial sections from each sample used. Staining was quantified using HALO software (Indica labs).

### 2.5 RT‐PCR

Frozen frontal cortex was prepared for RT‐PCR as previously described (Shaw et al., 2019). Briefly, tissue was homogenized in RLT Plus Buffer (Qiagen) using a Precelleys 24 dual‐tissue homogenizer (Bertin Technologies). RNA was then extracted using the Qiagen RNeasy Plus Mini Kit (Qiagen), as per the manufacturer's instructions. Total RNA was quantified with the Nanodrop One Spectrophotometer, with quality and purity of RNA determined using A260/A280 and A260/A230 ratios. Synthesis of cDNA was performed using the Superscript IV Reverse Transcription kit with random hexamers (Invitrogen) using a GeneAmp 9700 PCR Machine.

Primer sequences were designed and optimized for the guinea pig and are detailed in Table 2. The Fluidigm Juno and Biomark systems were utilized to obtain gene expression, as previously described (Crombie et al., 2023, 2021). According to the manufacturer's instructions, samples were preamplified using the PreAmp Master Mix. Primers were prepared at 0.5 pmol/μL in EVAgreen, and RT‐PCR was performed using the Biomark HD system. Plate consistency was ensured using a pooled brain sample as a calibrator. Fluidigm Real‐Time PCR Analysis Software was used for comparative ^ΔΔ^CT gene expression analysis and was normalized to housekeeper genes *ACTB*, *YWHAZ*, *UBE2D2*, and *TBP*.

**TABLE 2:** Guinea pig specific primers for used for real‐time polymerase chain reaction.

Gene ID	Protein	Forward primer	Reverse primer	Amplicon size (bp)
*DRD1*	Dopamine receptor D1	ACCTCCAGCATGGATGAGAC	TGACAGGAAACAGGCTGTCA	78
*DRD2*	Dopamine receptor D2	CCTGCCAAGCCAGAGAAGAA	GGGCATGGACTGGATCTCAAA	78
*TH*	Tyrosine hydroxylase	CCCTGGTTCCCACAAAAGGT	GAGAAACCCGGATGGTCCAG	95
*SLC6A3*	Dopamine transporter (DAT)	ACTTCCTGCTGTCCGTCATC	CCCCGCCATTTTTGTAGCAC	84
*RBFOX3*	RNA binding fox‐1 homolog 3 (NeuN)	CACAGACAGACAGCCAACCA	CGGAAGGGGATGTTGGAGAC	88
*PVALB*	Parvalbumin	AAGGATGGGGACGGCAAA	GGGTCCATCAGCTCTGCTTA	77
*CALB1*	Calbindin	CTGACTGAGATGGCCAGGTTA	CCCACACATTTTAACTCCCTGAAA	75
*SST*	Somatostatin	AAGCAGGAACTGGCCAAGTA	TGGGACAAATCTTCAGGTTCCA	92
*GAD 1* (*Sanchez‐Ramos*, *2005*)	Glutamate decarboxylase 1 (67 kDa)	AGC​TCG​CTA​CAA​GTA​CTT​CCC	TGT​GTT​CTG​AGG​TGA​AGA​GGA​C	83
*SLC1A2*	Excitatory amino acid transporter 3 (EAAT3)	CAC​AGT​CGT​CTC​CCT​GTT​GAA	CAG​GCC​CTT​CTT​GAG​AAC​CA	76
*MBP*	Myelin basic protein	ACCTCCTCCGTCTCAAGGAAA	GCTCTGCCTCCATAGCCAAA	66
*OLIG2*	Oligodendrocyte transcription factor	GCACTCATCCTGGGGACAA	CCGACGACGTGGATGATGAA	78
*NCAM1*	Neural cell adhesion molecule 1	TTGTTCCCAGCCAAGGAGAA	TGTCTTTGGCATCTCCTGCTA	78
*CSPG4*	Chondroitin sulfate proteoglycan 4	CTCCTCACCACCACCCTCAA	ACTCTTCAGCACAGCCCTCA	79
*GALC*	Galactosylceramidase	ACTTCCCGCCTTCTGGTAAA	AGGTTCAGTGCCATCTGTTGT	144
*SLC6A2*	Noradrenaline transporter (NET)	CTGACTCTGGGCCTTGACAG	AGAGCCAGAAGAAAGGTGCC	137
*GAD2*	GAD65 GABA synthesis	GGCGCCATCTCCAACATGTA	TGCCCTTCTCCTTGACCTCA	73
*SLC6A1*	GAT1 GABA transporter	AGCGCTGCTTCTCCAACTAC	ATTGCGCTCCCAAAACTCCA	77
*SLC6A11*	GAT3 GABA transporter	ATCATGCTCTGCTGCCTGAA	CATAAGCCATGAAGCCCAAGAC	82
*ACTB*	Housekeeper	TGCGTTACACCCTTTCTTGAC A	ACAAAGCCATGCCAATCTCAT	72
*YHWHAZ*	Housekeeper	GCTTCACAAGCAGAGAGCAA	CAGCAACTTCGGCCAAGTAA	76
*TBP*	Housekeeper	CAAGCGGTTTGCTGCTGTAA	CACCATCTTCCCGGAACTGAA	79
*UBE2D2*	Housekeeper	CAGTGCTGCGTGTTGTACATA	TGCTAGGAGGCAATGTTGGTA	77

*Note*: Primer sequences for detection of genes of interest in the guinea pig frontal cortex. Primer sequences are displayed from 5′−3′ for forward and reverse primers.

### 2.6 Statistical analysis

Data were analyzed using Prism v9.0 (Graphpad Software Inc.) and is presented as mean ± SEM with significance considered *p* < .05. Body weight and wellbeing scores were assessed over time by repeated measures two‐way ANOVA or mixed effects analysis. Single time point datasets were analyzed by one‐way ANOVA. Tukey corrections for multiple comparisons were performed when the overall analysis was *p* < .05. Data were split by sex.

## 3 RESULTS

### 3.1 Physical characteristics

Animal weights and organ‐body weight ratios are detailed in Table 3. At birth, preterm zuranolone males weighed significantly less than their term‐born counterparts (*p* = .02). Both preterm vehicle and zuranolone‐treated females weighed less than their term‐born counterparts at birth (*p* = .002 each). However, by corrected PND42, only preterm male vehicles weighed significantly less than their term‐born counterparts (*p* = .01). No changes were observed in growth rate for any of the groups. Preterm male vehicle animals displayed evidence of a potential brain‐sparing mechanism, as they had a significantly higher brain:body ratio than term‐born pups (*p* = .0027). Preterm male vehicle pups also displayed a significantly increased kidney:body ratio, whereas preterm female pups displayed a higher adrenal:body ratio (*p* = .01 and *p* = .0036, respectively). No changes were observed between preterm vehicle and zuranolone‐treated groups within each sex.

**TABLE 3:** Body and organ weights.

Sex	Delivery	Treatment	N	Birth Wgt	Postmortem body Wgt	Body weight growth %	Brain:Body (%)	Hippo:Brain (%)	Liver:Body (%)	Heart:Body (%)	Kidney:Body (%)	Adrenals:Body (%)
Male	Preterm	Vehicle	4	72.30 ± 10.36	287.31 ± 24.48*	427.6	1.07 ± 0.06*	4.21 ± 0.29	4.98 ± 0.32	0.49 ± 0.05	1.157 ± 0.04*	0.04 ± 0.005
		Zuranolone	3	60.80 ± 1.27*	306.02 ± 44.48	501.9	0.97 ± 0.08	3.75 ± 0.60	4.49 ± 0.33	0.48 ± 0.07	1.07 ± 0.01	0.034 ± 0.002
	Term	N/A	9	85.11 ± 3.65	333.63 ± 22.58	428.5	0.84 ± 0.03	3.86 ± 0.16	4.28 ± 0.16	0.42 ± 0.02	1.003 ± 0.02	0.035 ± 0.003
Female	Preterm	Vehicle	7	68.07 ± 4.81*	311.61 ± 19.06	462.2	0.97 ± 0.04	3.97 ± 0.35	4.17 ± 0.30	0.44 ± 0.01	1.02 ± 0.03	0.051 ± 0.002*
		Zuranolone	7	67.87 ± 2.987*	306.12 ± 6.17	455.5	0.97 ± 0.03	4.051 ± 0.26	4.22 ± 0.26	0.47 ± 0.06	1.01 ± 0.03	0.044 ± 0.007
	Term	N/A	11	90.20 ± 3.328	353.33 ± 10.50	396.5	0.860.03	4.347 ± 0.39	4.24 ± 0.16	0.44 ± 0.02	0.926 ± 0.02	0.037 ± 0002

*Note*: Mean ± SEM for body (in grams) and organ weights (ratio to body or brain weight) recorded upon tissue collection at corrected PND42. No differences between preterm groups within sex were observed.

Abbreviations: hippo, hippocampus; Wgt, weight.

*p < .05 compared to term within sex.

### 3.2 Growth and wellbeing

**FIGURE 1:**
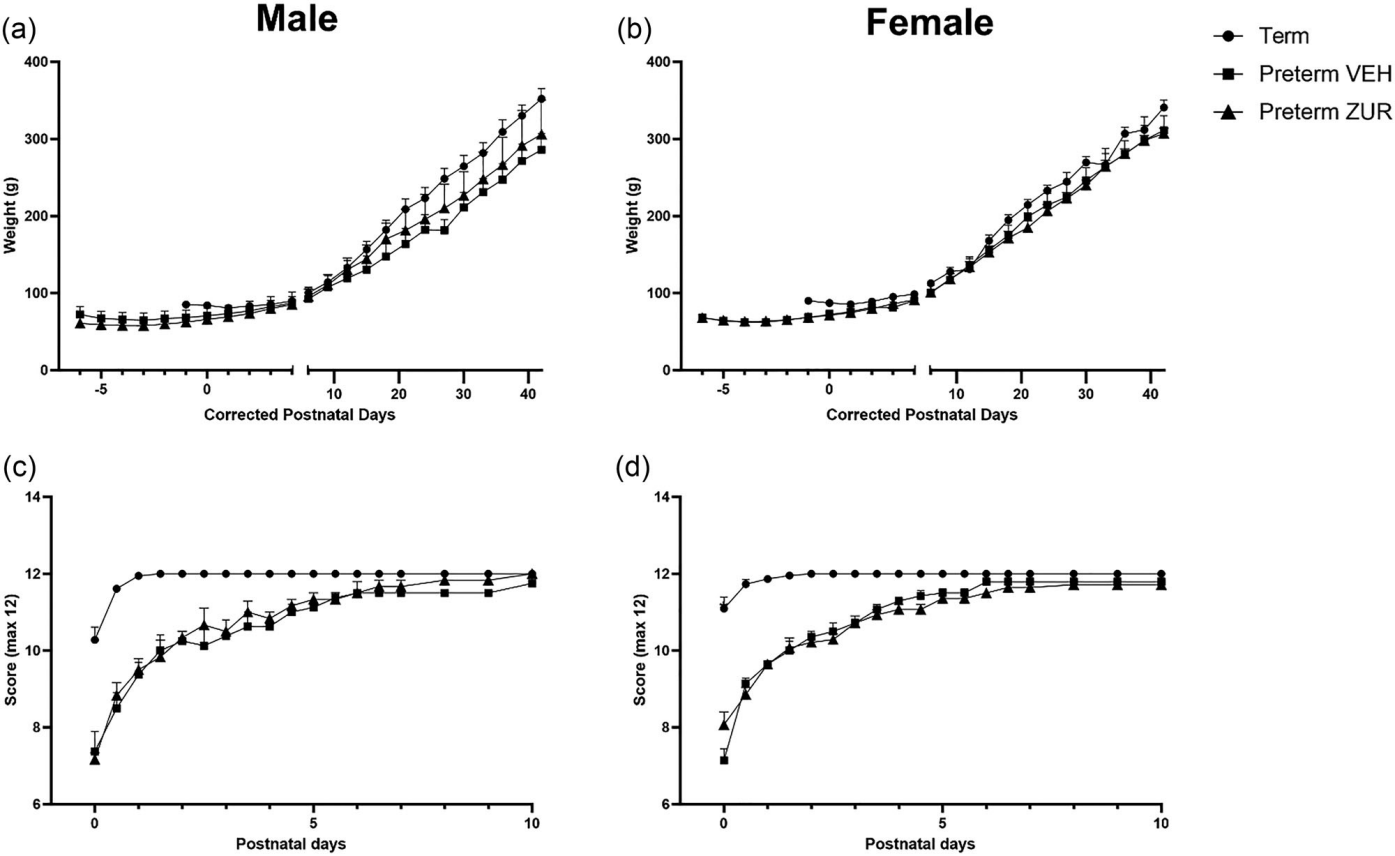
Body weight (a, b) and wellbeing scores (c, d) were recorded from birth until corrected postnatal day 42 in male (a, c) and female (b, d) term (circles; males *n* = 9, females *n* = 11), preterm vehicle (squares; male *n* = 4, females *n* = 7), and preterm zuranolone‐treated pups (triangles; male *n* = 3, females *n* = 7). Mean ± SEM.

Overall, body weight was found to change over time (*p* < .0001) but not between delivery and treatment groups within each sex (males *p* = .22; females *p* = .25) (Figure 1a,b).

Wellbeing scores were recorded every 2 h and averaged for 12 h totals, with a maximum score of 12. Overall, there was a significant effect of postnatal day and group on wellbeing scores in males and females (*p* < .0001) (Figure 1c,d). The preterm vehicle‐treated pup scores did not differ from the zuranolone‐treated pup scores for either males or females. However, the zuranolone‐treated preterm males scored significantly lower than term‐born males until PND6, while the vehicle‐treated preterm males scored significantly lower than term‐born males until PND10. The vehicle‐treated preterm‐born females had lower wellbeing scores than term‐born females until PND6, while the zuranolone‐treated preterm females‐scores were significantly lower than term‐born females until PND8.

### 3.3 Behavioral outcomes at early and late childhood equivalent ages

**FIGURE 2:**
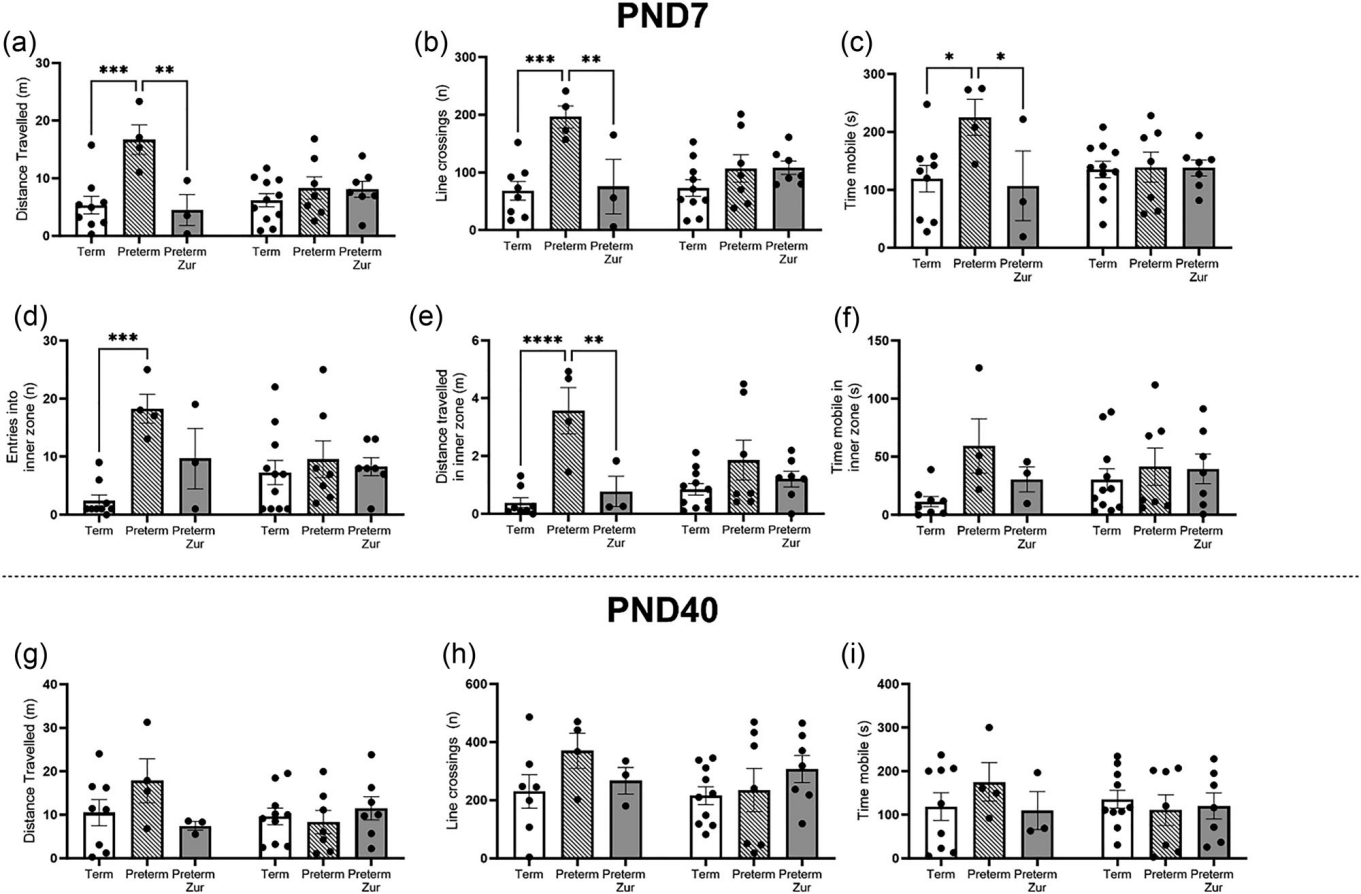
Open field arena exploration outcomes for term control (black bars; males *n* = 9, females *n* = 11), vehicle‐treated preterm (striped bars; males *n* = 4, females *n* = 7), and zuranolone‐treated preterm pups (gray bars; males *n* = 3, females *n* = 7) at corrected postnatal day (PND) 7 (a–f) and 40 (g–i). Parameters measured included distance traveled (a, g), line crossings (b, h), time mobile in the total arena (c, i), in addition to entries into (d), distance traveled (e), and time mobile in the inner zone (f). Data presented as mean ± SEM, with significance at *p < .05, **p < .01, ***p < .001, and ****p < .0001.

**FIGURE 3:**
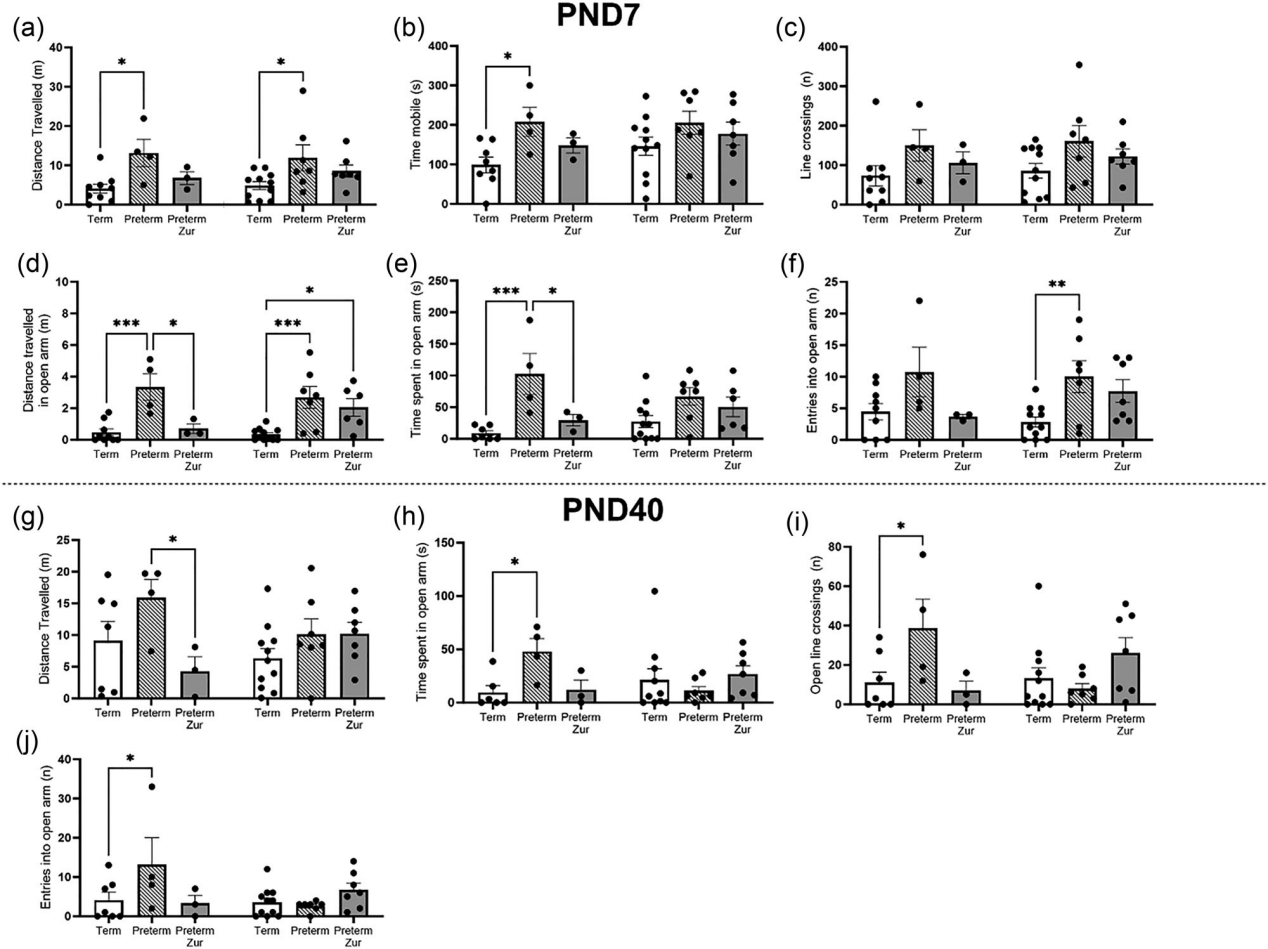
Elevated plus maze outcomes for term control (black bars; males *n* = 9, females *n* = 11), vehicle‐treated preterm (striped bars; males *n* = 4, females *n* = 7), and preterm pups that received zuranolone therapy (gray bars; males *n* = 3 males, females *n* = 7) at corrected postnatal day (PND) 7 (a–f) and 40 (g–k). Parameters measured included distance traveled (a, g), line crossings (b, h), and time mobile in the total maze (c, i), in addition to entries into (d), distance traveled (e), and time mobile in the inner zone (f). Data presented as mean ± SEM, and significance *p < .05, **p < .01, ***p < .001, and ****p < .0001.

Behavioral outcomes were assessed using both the open field arena (Figure 2) and elevated plus maze (Figure 3), at PND7 to represent early childhood and PND40 to represent late childhood.

#### 3.3.1 Open field

In the open field arena, male vehicle‐treated preterm pups overall displayed a hyperactive phenotype at PND7. This was evidenced through a significant increase in distance traveled in the arena (Figure 2a; *p* = .01), line crossings (Figure 2b; *p* = .03), time mobile (Figure 2c; *p* = .01), entries into the inner zone (Figure 2d; *p* = .0004), as well as distance traveled in the inner zone (Figure 2e; *p* < .0001) compared to term‐born male pups. Interestingly, male zuranolone‐treated preterm pups’ activity did not differ from term‐born pups, and furthermore, they demonstrated significantly lower distance traveled (Figure 2a; *p* = .001), line crossings (Figure 2b; *p* = .004), time mobile (Figure 2c; *p* = .04), and distance traveled in the inner zone (Figure 2e; *p* = .004), compared to preterm vehicle pups. These changes were no longer observed at PND40 (Figure 2g–i). No changes were observed in females at either age.

#### 3.3.2 Elevated plus maze

At PND7, vehicle‐treated preterm‐born males demonstrated a significant increase in distance traveled (Figure 3a; *p* = .02) and time mobile (Figure 3b; *p* = .04) in the maze, as well as distance traveled (Figure 3d; *p* = .0005) and time spent (Figure 3e; *p* = .0005) in the open arm compared to term‐born male pups. Importantly, zuranolone treatment significantly reduced distance traveled (Figure 3d; *p* = .01), and time spent (Figure 3e; *p* = .02) in the open arm compared to vehicle‐treated preterm male pups. Term‐born and zuranolone‐treated preterm‐born male pups did not differ for any parameter.

At PND7, vehicle‐treated preterm‐born females also demonstrated significantly increased distance traveled (Figure 3a; *p* = .01) in the maze and entries into the open arm (Figure 3f; *p* = .009), compared to term controls. Vehicle and zuranolone‐treated preterm females also had an increased distance traveled in the open arm compared to term controls (Figure 3d; *p* = .0004, *p* = .01).

At PND40, significant behavioral changes were only evident in males. Vehicle‐treated preterm males continued to spend significantly greater time in the open arm of the maze (Figure 3h; *p* = .04), increased open arm line crossings (Figure 3i; *p* = .04), entries into the open arm (Figure 3j; *p *= .03) and time spent in the open arm (Figure 3k; *p* = .04). There was a significant reduction in distance traveled by the preterm zuranolone‐treated pups compared to those that had received vehicle (Figure 3 g; *p* = .04). Again, term‐born and zuranolone‐treated preterm male pups were not found to differ nor were there any differences between the female pups at PND40.

#### 3.3.3 Acoustic startle reflex

No significant differences were observed in ASR, PPI, habituation, or latency at either PND8 or PND41 for either preterm or term, treatment or sex (data not presented).

### 3.4 Immunostaining of myelin in the frontal lobe

**FIGURE 4:**
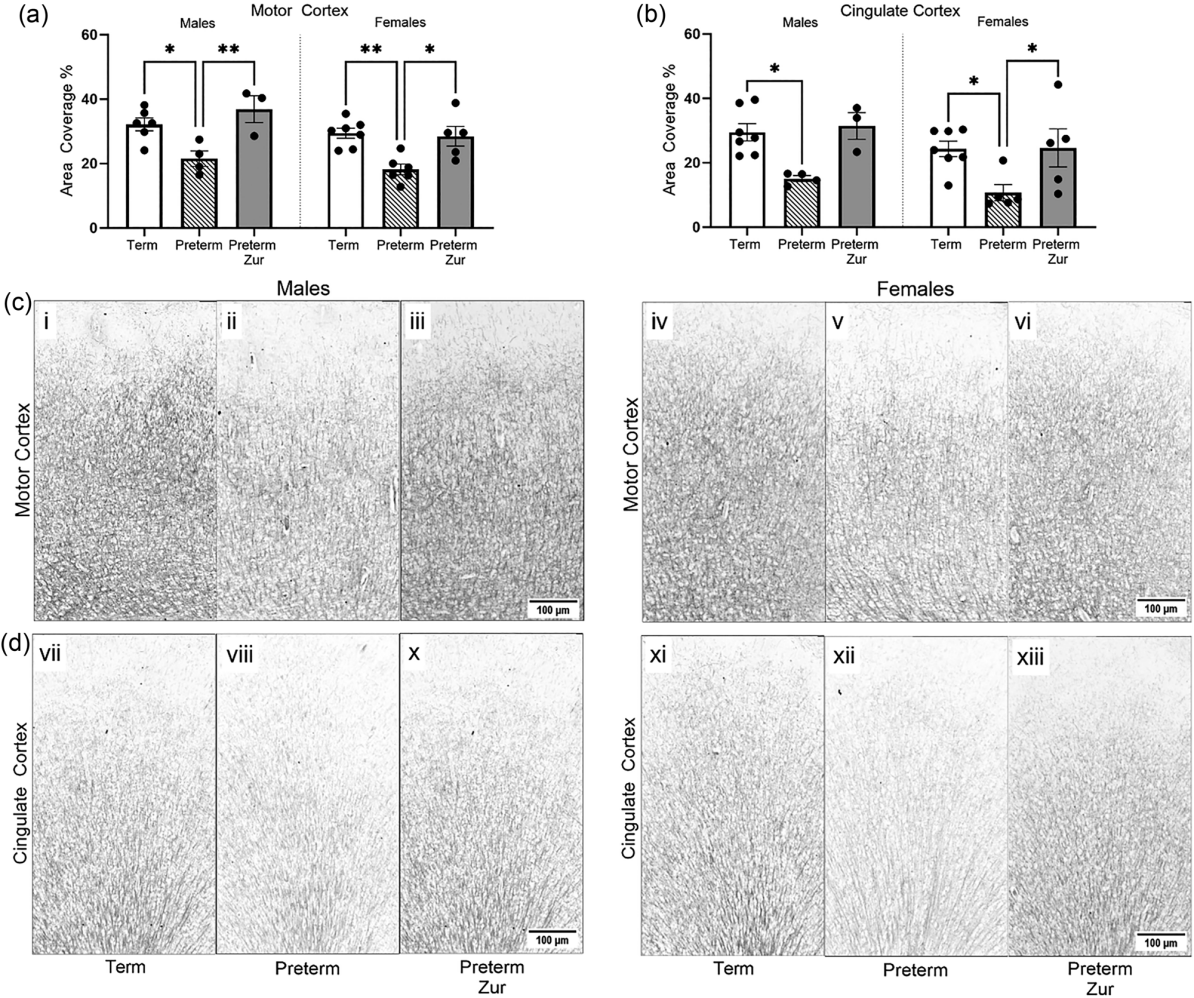
Myelin basic protein (MBP) immunostaining in the motor (a) and cingulate cortex (b) of term control (black bars; males *n* = 9, females *n* = 11), preterm vehicle (striped bars; males *n* = 4 males, females *n* = 7), and preterm zuranolone‐treated pups (gray bars; males *n* = 3 males, females *n* = 7). Representative photomicrographs of MBP immunostaining of the motor cortex (c) and cingulate cortex (d) (i, vi = term males; iv, xi = term females; ii, viii = preterm vehicle‐treated males; v, xii = preterm vehicle‐treated females; iii, x = preterm zuranolone‐treated males; vi, xiii = preterm zuranolone‐treated females). Data presented as mean ± SEM with significance at *p < .05 and **p < .01.

Immunostaining of MBP was assessed in the motor and cingulate cortices of the frontal lobe (Figure 4). Both male and female preterm pups that received vehicle had significantly less immunostaining for MBP in both the motor (Figure 4a; *p* = .01, *p* = .004) and cingulate cortex (Figure 4b; *p* = .03, *p* = .03) compared to term. Importantly, preterm‐born male and female zuranolone‐treated pups had significantly increased immunostaining in the motor cortex compared to their preterm vehicle counterparts (Figure 4a; *p* = .03, *p* = .03), with females also displaying this increase in the cingulate cortex (Figure 4b; *p* = .04) and males displaying a trend to increase (Figure 4b; *p* = .058). There was no difference in the staining between term and zuranolone‐treated preterm pups in either area.

### 3.5 Relative expression of key neurodevelopmental genes in the frontal cortex

#### 3.5.1 Oligodendrocyte lineage and neuronal markers

**FIGURE 5:**
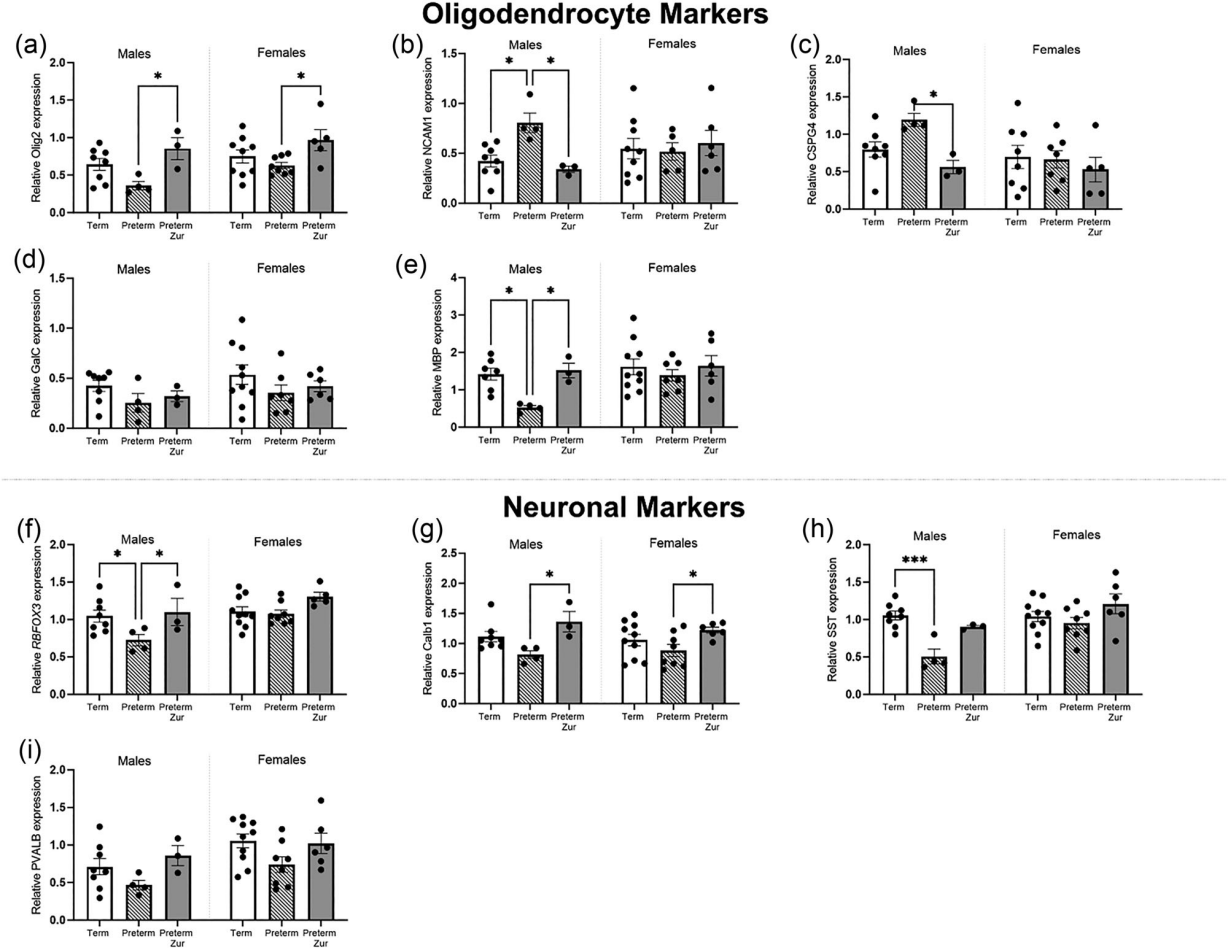
Relative mRNA expression of key oligodendrocyte [(a) *Olig2*, (b) *NCAM1*, (c) *CSPG4*, (d) *GalC*, (e) *MBP*] and neuronal [(f) *RBFOX3*, (g) *Calb1*, (h) *SST*, (i) *PVALB*] markers in the frontal cortex obtained from term control (black bars; males *n* = 9, females *n* = 11), preterm vehicle treated (striped bars; males *n* = 4, females *n* = 7), and preterm zuranolone‐treated pups (gray bars; males *n* = 3, females *n* = 7). Data presented as mean ± SEM with significance at *p < .05, **p < .01, ***p < .001, and ****p < .0001.

The mRNA expression of key oligodendrocyte lineage and neuronal markers in the frontal cortex was examined at PND42 (Figure 5). Oligodendrocyte markers that were quantified included oligodendrocyte transcription factor (*Olig2*), a marker of the entire oligodendrocyte lineage; neural cell adhesion molecule (*NCAM1*), a marker of early oligodendrocyte development that is expressed in early oligodendrocyte pre‐progenitor cells (OPPs); neuron‐glial antigen 2 (NG2; *CSPG4*), a marker of oligodendrocyte progenitor cells (OPCs); galactocerebroside (*GalC*), a marker of immature oligodendrocytes and; myelin basic protein (*MBP*), a marker of mature, myelinating oligodendrocytes.

Interestingly, male preterm pups that received vehicle had a significant increase in *NCAM1* compared to term born (Figure 5b; *p* = .04), which was then significantly reduced following zuranolone treatment (*p* = .04). Similarly, *MBP* expression was significantly reduced in male preterm vehicle‐treated pups compared to term controls (Figure 5e; *p* = .02) and again this was restored with zuranolone treatment (*p* = .04). Zuranolone‐treated male and female pups had a significant increase in *Olig2*, compared to vehicle‐treated preterm animals (Figure 5a; *p* = .01, *p* = .03, respectively). Similarly, there appeared to be an increase of *CSPG4* in male preterm vehicles compared to control but it did not reach significance (Figure 5c; *p* = .1), however, there was significantly reduced expression following zuranolone treatment (*p* = .04), with levels restored to control. No changes were observed in *GalC* expression.

The neuronal markers that were quantified included RNA binding protein fox‐1 homolog (*RBFOX3*), a marker of overall neurons, as well as three key GABAergic interneurons, calbindin (*Calb1*), somatostatin (*SST*), and parvalbumin (*PVALB*). Preterm‐born males had a significant reduction of RBFOX3 expression, which was significantly returned to control levels with zuranolone treatment (Figure 5f; *p* = .03, *p* = .04. Zuranolone treatment also increased the expression of *Calb1* in both sexes when compared to preterm vehicle counterparts (Figure 5g; *p* = .02, *p* = .04). Interestingly, only male preterm vehicle pups had a significant reduction of *SST* expression compared to term controls (Figure 5h; *p* = .0007), which was normalized to control levels following zuranolone treatment. No changes were observed for *PVALB* expression.

#### 3.5.2 Neurotransmitter pathway markers

**FIGURE 6:**
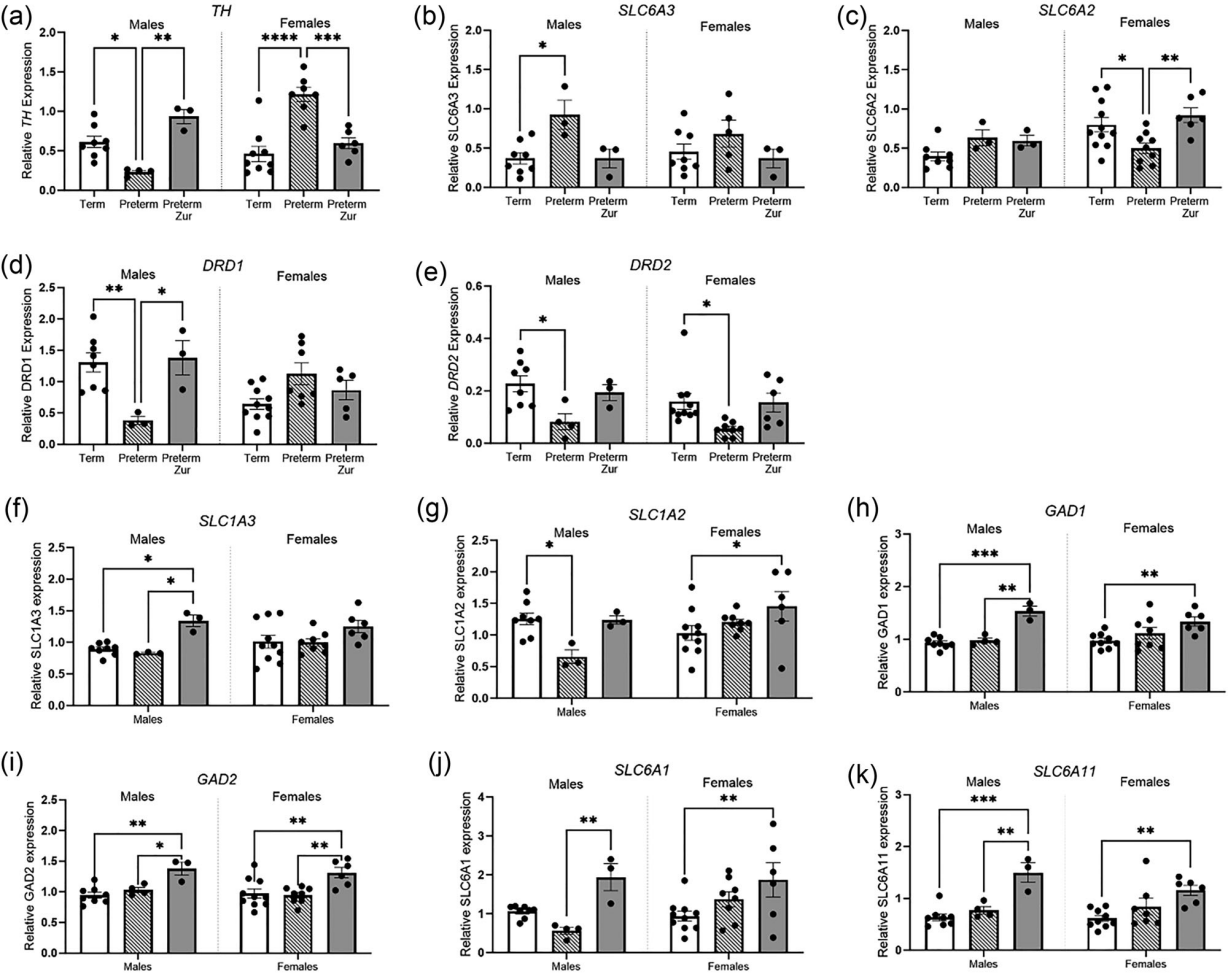
Relative mRNA expression of dopaminergic [(a) *TH*, (b) *SLC6A3*, (c) *SLC6A2*, (d) *DRD1*, (e) *DRD2*], glutamatergic [(f) *SLC1A3*, (g) *SLC1A2*], and GABAergic [(h) *GAD1*, (i) *GAD2*, (j) *SLC6A1*, (k) *SLC6A11*] pathway components in the frontal cortex obtained from term control (black bars; males *n* = 9, females *n* = 11), preterm vehicle treated (striped bars; males *n* = 4 males, females *n* = 7), and preterm zuranolone‐treated pups (gray bars; males *n* = 3, females *n* = 7). Data presented as mean ± SEM with significance at *p < .05, **p < .01, ***p < .001, and ****p < .0001.

The mRNA expression of several key components of the dopaminergic, GABAergic, and glutamatergic pathways were quantified. Tyrosine hydroxylase (*TH*; rate limiting enzyme in dopamine synthesis) was significantly reduced in male preterm pups that received vehicle when compared to both term (Figure 6a; *p* = .04) and zuranolone‐treated pups (*p* = .0001). There was no difference between term and zuranolone‐treated male preterm pups. Conversely, female preterm pups that received vehicle had significantly increased expression of *TH* (*p* < .0001); however, this was again significantly normalized with treatment (*p* = .0001). Expression of the dopamine transporter DAT (*SLC6A3*; removal of dopamine from synaptic cleft) was significantly increased in male preterm pups that received vehicle (Figure 6b; *p* = .03), while zuranolone‐treated pups were not changed, when compared to term controls. There were no significant changes observed for females. Interestingly, for the noradrenaline transporter NET (*SLC6A2;* removal of noradrenaline from synaptic cleft), there were no changes observed in males. Preterm vehicle‐treated females, however, had a significant reduction in *SLC6A2* (Figure 6c; *p* = .03), which was significantly increased by zuranolone treatment (*p* = .007). Dopamine exerts its actions through two main families of receptors, the dopamine D1‐like (*DRD1)* and D2‐like (*DRD2*) families. Male preterm vehicle‐treated pups had a significant reduction in *DRD1* expression (Figure 6d; *p* = .0054), which again was significantly increased following zuranolone treatment (*p* = .01). Both male and female preterm vehicle pups had a significant reduction in *DRD2* expression compared to control animals (Figure 6e; *p* = .02, *p* = .04), with levels remaining the same as in control term pups following zuranolone treatment.

Excitatory amino acid transporters EAAT1 (*SLC1A3*) and EAAT2 (*SLC1A2*) comprise two of the major glutamate reuptake transporters that remove glutamate from the synaptic cleft. Preterm male zuranolone‐treated pups had significantly increased *SLC1A3* compared to both term control (Figure 6f; *p* = .01) and preterm vehicle‐treated pups (*p* = .02). Meanwhile, preterm male vehicle pups had a significant reduction in *SLC1A2* (Figure 6g; *p* = .03), with levels remaining unchanged from term levels following zuranolone treatment. In females, zuranolone treatment in preterm animals significantly increased expression compared to control pups (Figure 6g; *p* = .04).

Glutamate decarboxylase 1 (GAD67; *GAD1)* and 2 (GAD65; *GAD2*) are enzymes responsible for synthesizing GABA from glutamate. *GAD1* was significantly increased following zuranolone treatment in male preterm pups compared to term control (Figure 6h; *p* = .0002) and vehicle‐treated pups (*p* = .002). Expression was also significantly increased in female preterm pups that received vehicle compared to term controls (Figure 6h; *p* = .003). Similarly, *GAD2* expression was significantly increased in both male (Figure 6i; *p* = .003, *p* = .04) and female (*p* = .002, *p* = .001) zuranolone‐treated female pups, compared to both term control and vehicle‐treated preterm animals. GABA transporters 1 (GAT1; *SLC6A1*) and 3 (GAT3; *SLC6A11*) move GABA from the synaptic cleft to the presynaptic neuron or surrounding glial cells to be recycled. *SLC6A1* was significantly increased in zuranolone‐treated male pups compared to preterm vehicle‐treated males (Figure 6j; *p* = .007) and was also increased in female zuranolone‐treated female pups compared to term females (*p* = .007). Similarly, *SLC6A11* expression was significantly increased in preterm zuranolone‐treated male pups compared to term controls (Figure 6k; *p *= .0001) and preterm males that received vehicle (*p* = .003). Furthermore, *SLC6A11* was increased in female zuranolone‐treated preterm‐born pups compared to those that received vehicle (Figure 6k; *p* = .001). There were no other significant changes identified.

## 4 DISCUSSION

The overarching key finding in this study was that treatment of preterm guinea pig neonates with zuranolone in the immediate postnatal period prevented marked behavioral changes. Preterm‐born offspring exhibited a hyperactive phenotype, which continued through development until at least the equivalent of late childhood and was particularly evident in male offspring. These offspring also had reductions in myelin protein in two key areas in the frontal cortex. Preterm‐born males also exhibited hypodopaminergic expression changes, which is consistent with what was previously observed in our guinea pig model (Moloney et al., 2024). When treated with zuranolone in the immediate postnatal period, measures of behavioral abnormalities were restored to levels observed in term controls. Additionally, treatment appeared to restore myelination levels to that of term controls, as well as reversing neurotransmitter mRNA expression abnormalities. These preterm associated deficits are consistent with an ADHD phenotype, and treatment with zuranolone may represent a novel treatment option for the prevention of this long‐term disorder.

We have previously shown that preterm‐born guinea pigs exhibit a hyperactive phenotype, which continues into late childhood (Shaw et al., 2019, 2016). Clinically, it is well understood that preterm‐born infants have an increased risk of life long neurobehavioral disorders, one of the most prevalent being ADHD. ADHD is characterized by symptoms of inattention and hyperactivity, and typically presents during childhood (Rommel et al., 2017; Wilens & Spencer, 2010). In this study, preterm males displayed a strong hyperactive phenotype at the equivalent of early childhood in the open field arena. While the data indicated this did not persist at the older age, the size of the arena may not have been sufficient or suitable for the substantial increase in the size of the animals. The elevated plus maze, however, revealed hyperactive behavior at both ages assessed. In both of these behavioral assessments males were particularly affected, which is consistent with clinical observations indicating males have a higher prevalence to ADHD (Stibbe et al., 2020). It is, however, worth noting that preterm birth is associated with both the hyperactive/impulsive and the inattentive subtype of ADHD (Montagna et al., 2020). Due to the neonatal time point that the behavioral assays were conducted at, it was difficult to perform tasks that require learning as there is a limited window of time to capture early childhood behavior. The difficulty in assessing inattention is a limitation of the model and further research is warranted to explore other behavioral assessments that may be more suitable for identifying inattentive behavior, such as the attentional set‐shifting task. Acoustic startle assessment has shown that reduced habituation, pre‐pulse inhibition, and dysregulation of gating responses have been associated with schizophrenia (Akdag et al., 2003; Braff et al., 1992; Mena et al., 2016). In this study, we found no significant differences in any of these parameters suggesting that the preterm‐born offspring developed a largely hyperactive phenotype rather than a schizophrenia‐like phenotype at the ages assessed. Additionally, there were no differences observed with corrected weight between preterm‐ and term‐born pups, further highlighting that there were no observed signs of growth restriction or other intrauterine disruptions that would be causing the changes observed. Due to the difficulty in obtaining blood samples in the neonates, quantification of blood gas levels in the offspring was not performed and as such is a limitation of this study. We accounted for this by using the scoring system to broadly encompass obvious signs of hypoxia; however, future work would be warranted in exploring blood gas levels following preterm birth further. Preterm‐born male and female vehicle‐treated offspring had significant reductions in frontal cortex myelination, which were not found after zuranolone treatment. The deficits seen after preterm birth occurred in both the motor and cingulate cortex, which are two critical regions that play roles in the regulation of attentional control. The motor cortex contains a dense projection of dopaminergic neurons, and through fMRI studies is shown to be activated during tasks related to sensory attention stimulation (Johansen‐Berg & Matthews, 2002). This myelination is crucial, as studies have shown that deficits in myelin in this region are associated with impaired learning and abnormal spontaneous activity (Kato et al., 2020). Similarly, the cingulate cortex functions to modulate external sensory input and maintain attention (Wu et al., 2017). Morphological changes have been observed in the cingulate cortex in patients with ADHD, particularly involving reductions in overall volume in this region (Si et al., 2021) as well as reduced connectivity (Vogt, 2019). This may be explained by reduced white matter integrity in this region, as it is well established that children with ADHD have white matter abnormalities in this region which have been linked with symptoms of inattention and impulsivity (Chiang et al., 2015, 2016, bib16; King et al., 2015; Tung et al., 2021). The present finding indicates that treatment with zuranolone enhances myelination, which may explain the marked normalization of the measured behavioral parameters in the treated pups. Further behavioral assessments at older ages, such as late adolescence, may be useful in clarifying the behavior of these preterm‐born offspring.

Close examination of the key genes associated with myelination and neurons showed that there were several marked alterations in gene expression found in the preterm pups that received vehicle treatment. Myelin‐producing oligodendrocytes require progression through a developmental lineage with only mature cells able to produce myelin (Bradl & Lassmann, 2010). In this study, we examined gene expression representing all stages of the oligodendrocyte lineage, and one of the major findings was that preterm‐born males displayed what appeared to be an arrested maturation of oligodendrocytes. This observation further validates our preterm model, as clinically preterm infants have been shown to have an arrested maturation of oligodendrocytes (Back et al., 2002). Preterm male animals that received vehicle had increased expression of earlier oligodendrocyte markers in the lineage, and reductions in *MBP*. Importantly, zuranolone treatment appeared to normalize this expression back to control term levels, highlighting the beneficial effects of this drug on promoting oligodendrocyte maturation. Interestingly, females did not display the same changes in mRNA expression, however, also showed reductions in myelination, which may indicate a potential different mechanism involved in the arrest of myelination. This may involve differences in hormonal levels, as previous studies have indicated that androgens contribute to differences in demyelination and repair observed between sexes (Bielecki et al., 2016; Hussain et al., 2013).

In addition to changes in oligodendrocyte gene expression, we also found reductions in overall neuronal gene expression, particularly somatostatin (*SST*) expression. A previous study found that somatostatin activity was associated with risk‐taking behavior and hyperactivity, particularly with male mice (Brockway et al., 2023). Somatostatin has been proposed to “fine‐tune” prefrontal activity and therefore may be modulating behavioral abnormalities observed in this study. Treatment with zuranolone was able to prevent deficits observed in the mRNA expression of this interneuron and promote gene expression of another GABAergic interneuron (*Calb1*), indicating that the observed neuroprotective action may involve increasing GABAergic inhibition.

Neurobehavioral disorders involve disruptions to key neurotransmitter pathways, including the dopaminergic, GABAergic, and glutamatergic systems. There is currently limited knowledge relating to mechanisms by which preterm birth affects these neurotransmitter pathways. These changes may be a key driver behind the increased risk of neurobehavioral disorders seen following preterm birth. In this study, preterm birth appeared to affect the dopaminergic pathway in a sex‐dependent manner. Preterm‐born males showed a reduction in dopamine synthesis mRNA expression (*TH*) and an increase in dopamine reuptake (*SLC6A3*), overall indicating a hypodopaminergic phenotype. This may be potentiated by reductions in the key dopamine receptors *DRD1* and *DRD2*. Reduced dopamine in the frontal cortex is associated with ADHD, as dopamine action is one of the key modulators that facilitates attentional control in the frontal cortex (Kollins & Adcock, 2014). Interestingly, preterm female offspring displayed an opposite effect with what appears to be an increase in dopamine synthesis expression, and a reduction in noradrenaline reuptake. This could indicate an increase in noradrenergic activity, which is associated with anxiety‐like symptoms in the frontal cortex (Bouras et al., 2023). Despite these opposing observations, treatment with zuranolone restored mRNA expression of this pathway back to a term‐born phenotype. This suggests that zuranolone may not be targeting the dopamine pathway specifically, but rather reducing the effects that may be associated with these alterations. While we have shown strong evidence of neurotransmitter pathway disruption at the mRNA level, it would be useful to assess modulation by also examining the protein and activity of these key components.

The neuroprotective effects of zuranolone may be mediated through reduction of glutamatergic activity as well as the promotion of the protective GABAergic inhibitory tone. This could be interpreted as mimicking the environment of the developing brain and the exposure during development. In both sexes, zuranolone treatment increased the expression of two key glutamatergic reuptake transporters (*SLC1A3* and *SLC1A2*) responsible for majority of the glutamate reuptake from the synaptic cleft (Magi et al., 2019). Zuranolone also increased expression of *GAD1* and *GAD2*, which catalyze the decarboxylation of glutamate to GABA, therefore increasing synthesis of GABA, as well as the expression of the GABA transporters 1 and 2, which may increase the recycling of GABA. These findings suggest there are multiple actions occurring with zuranolone treatment that may be affecting longer term changes in excitability.

Currently, breastfeeding while taking zuranolone for PPD is contraindicated due to the unknown and possible negative effects on the neonate (Prasad & Allely, 2024). This study has demonstrated the benefits to long term behavioral outcomes and neurodevelopment following direct zuranolone exposure. Recent research has concluded that functional MRI scanning of fetal brains may be a predictor of preterm birth, and that abnormal neurodevelopment may be occurring in utero (Miglioli et al., 2024). Investigating neurodevelopmental predictors in utero and observing how those change ex utero would provide an interesting link to how these damages are occurring and how the development of neurobehavioral conditions may change as a result. Utilizing this, future work is warranted to explore if zuranolone may be used as a treatment during gestation, in high‐risk cases of preterm birth. Administration of synthetic progestogens has been shown to reduce the incidence of preterm birth (Doggrell, 2003; Sanchez‐Ramos, 2005), and research is ongoing to ascertain if neurosteroids such as allopregnanolone may have similar effects. While this may be useful in preventing preterm birth, it may also prove to be beneficial for neuroprotection for the developing infant. Additionally, it is important to recognize that preterm birth that is associated with severe maternal conditions, such as chorioamnionitis, infection, and fetal growth restriction, are likely to more severe neurodevelopmental outcomes (Villar et al., 2024; Villar et al., 2021). As there are currently no available treatments for neuroprotection from these conditions, the use of postnatal neurosteroid therapy with zuranolone may offer a greater benefit than what we have observed in this study. Future work further investigating more severe phenotypes would be beneficial.

It is also important to consider the effects that zuranolone may be having peripherally. We observed no differences in weight between zuranolone‐treated and vehicle‐treated pups, indicating that despite the increased inhibitory effects at the mRNA level, the drug does not appear to be causing sedatory effects that interfere with feeding and weight gain. These observations are further supported by the consistently similar if not better well‐being scores between zuranolone‐treated preterm offspring compared to the preterm pups that received vehicle. Despite preterm zuranolone male pups having a significantly lower birth weight than that of the term controls, this difference was restored by PND42, unlike preterm vehicles who remained smaller. Brain‐sparing also appeared to occur in the zuranolone‐treated preterm males as evidenced through the increased brain:body ratio. Notably, there were no effects of zuranolone treatment in growth of other organs.

In conclusion, this study is the first to show that zuranolone treatment for preterm‐born offspring in the immediate postnatal period has long‐term benefits in restoring dysregulated behavior, myelination, and mRNA expression of neurotransmitter pathways. This study has also highlighted the neuroprotective actions of zuranolone therapy and demonstrated marked benefits the treatment may have on outcomes. Overall, the work shows that zuranolone may be the first medication for improving neurodevelopment that can reduce the risk of serious behavioral disorders in children born preterm. Future work is warranted to examine the effects of this neuroprotective therapy past the late childhood stage to see if these neuroprotective effects continue into adolescence and adulthood, for prevention of life‐long conditions such as ADHD.

## AUTHOR CONTRIBUTIONS

**Roisin A. Moloney**: Conceptualization; investigation; writing—original draft; methodology; writing—review and editing; formal analysis; data curation; project administration. **Hannah K. Palliser**: Funding acquisition; writing—review and editing; supervision; resources; methodology. **Carlton L. Pavy**: Methodology; resources. **Julia C. Shaw**: Funding acquisition; methodology; writing—review and editing; supervision; resources. **Jonathan J. Hirst**: Resources; supervision; project administration; writing—review and editing; funding acquisition; methodology.

### PEER REVIEW

The peer review history for this article is available at https://publons.com/publon/10.1002/brb3.70009

## Data Availability

The data that support the findings of this study are available from the corresponding author upon reasonable request.
